# Technology Use Among Older Adults and Their Caregivers: Cross-Sectional Survey Study

**DOI:** 10.2196/50759

**Published:** 2024-05-01

**Authors:** Shinduk Lee, Marcia G Ory, Deborah Vollmer Dahlke, Matthew Lee Smith

**Affiliations:** 1 College of Nursing, University of Utah, Salt Lake City, UT, United States; 2 Department of Environmental and Occupational Health, Texas A&M University, College Station, TX, United States; 3 Center for Community Health and Aging, Texas A&M University, College Station, TX, United States; 4 DVD Associates, LLC, Austin, TX, United States; 5 Department of Health Behavior, Texas A&M University, College Station, TX, United States

**Keywords:** technology, caregiving, social determinants of health, health disparities, disparity, disparities, caregiver, caregivers, carers, technology use, usage, gerontology, geriatric, geriatrics, older adult, older adults, elder, elderly, older person, older people, ageing, aging, cross-sectional, survey, surveys, computer use, device, devices, adoption, dyad, dyads

## Abstract

**Background:**

Informal caregivers are called upon to provide substantial care, but more needs to be known about technology use among older adult and caregiver dyads.

**Objective:**

This study described technology use among older adults and their caregivers, explored potential correlates of technology use, and highlighted implications for practice.

**Methods:**

A cross-sectional survey was conducted among unpaid caregivers of older adults (n=486). Primary outcomes were self-reported technology (devices and functions) use among caregivers and their oldest care recipient. The concordance of technology use among caregivers and care recipients was also examined. Multivariable regression models were conducted separately for caregivers and care recipients.

**Results:**

Greater proportions of caregivers used all examined technologies, except for the medication alerts or tracking function, than care recipients. Caregivers used an average of 3.4 devices and 4.2 functions, compared to 1.8 devices and 1.6 functions used by their care recipients. Among caregivers, younger age, higher income, and higher education were associated with more technology use (*P*<.05). Among care recipients, younger age, not having cognitive dysfunction, and caregiver’s technology use were associated with more technology use (*P*<.05).

**Conclusions:**

Understanding technology use patterns and device adoption across diverse caregiver and care recipient populations is increasingly important for enhancing geriatric care. Findings can guide recommendations about appropriate technology interventions and help providers communicate and share information more effectively with patients and their caregivers.

## Introduction

Aging is occurring in most parts of the world [1]. Driven by the large baby boomer generation, the American population is aging rapidly [2], with 1 in 4 Americans estimated to be 65 years or older by 2060 [3]. Although healthy aging may be the “new normal” for some [4], normative age-related decline in physical and cognitive functions exists and often results in the need for assistance with daily household and self-care activities. Among 53 million American adult informal caregivers, nearly 42 million cared for adults aged 50 years or older [4,5]. Despite the many hours devoted to informal caregiving [4,5], many older adults face adverse consequences of unmet needs for assistance with daily activities [6,7]. In addition to inadequate caregiving resources for older Americans, the negative impacts of caregiving on caregivers’ health and quality of life have raised significant programmatic and policy concerns [8-10].

An array of innovative technology solutions exists to support older adults’ health, independence, and quality of life, enabling them to age in place [11-14]. These technologies also support caregivers and enhance caregiving for older adults in areas such as fall alert notifications, social supports, communication, and medication scheduling [15,16]. However, older age is frequently considered a prominent factor associated with diminished interest in, and adoption of, technology [17-19]. This can be attributed to unique barriers associated with older age, including the lack of experiences or familiarity and declining physical, cognitive, and sensory functions [20,21]. Although recent data suggest a narrowing of the age-based gap in the digital divide [5,22,23], data also show that older adults may not use technology to its full potential [5].

The latest national surveillance data showed that about 24% of baby boomers provided informal care [24], with older caregivers tending to provide care for care recipients at similar or older ages [25]. The existence of any age-related difficulty in accessing or using technology is relevant for understanding health technology use among older adults in need of care, as well as their older caregivers who can use technology for caregiving. Although abundant literature discusses technology use among older adults or caregivers [5,25-27], few studies have examined technology use among both older adults and their caregivers [28,29] and the potential relationship between older adults and their caregivers’ technology use [16].

The relationship between older adults and their caregivers is interdependent, extending to their use of technology. Knowing the pattern of technology use in older adults and their caregivers can inform the development of technology-based interventions that are accessible and usable to the aging community. For example, a qualitative study involving patients with type 1 diabetes and their spouses revealed that continuous glucose monitoring technology can enhance spousal engagement in diabetes care, yet it may also introduce sources of tension within the relationship [30]. The qualitative study provided preliminary data to guide the development of a technology-based intervention, called SHARE plus [31]. In another recent study, Shih et al [32] focused on the different types of digital devices and categories of smartphone functions used by caregivers and care recipients compared to those with no caring roles. Shih et al [32] developed a health-related smartphone app for older adults and their caregivers, and their recent work was conducted to improve the design of their smartphone app.

Our study sought to further our understanding of how the use of technology by caregiver and care recipient dyads can guide intervention outlets (eg, digital platforms) and support efficient deployment of the technology interventions, including expanded access and use of technology functions. Therefore, technology use was broadly defined in this study to enable research into the use of diverse types of devices and functions. Devices and functions represent different aspects of technology use. Devices encompass the equipment or hardware of technology, whereas functions pertain to the specific tasks one can perform using technology. For instance, an individual may possess a smartphone, tablet, and computer but only use them for internet browsing. In contrast, another person with access solely to a smartphone may use it for various functions such as email and texting, internet browsing, web-based banking, and more. Analyzing devices and functions independently can offer more precise insights to inform future technology-based interventions for the aging community.

Our key aims were (1) to describe the use of various technologies (ie, both devices and functions) for caregivers and older adult care recipients; (2) to compare technology use among caregivers and older adult care recipients; and (3) to examine potential correlates of caregivers’ and older adult care recipients’ technology use. A study by Lindeman et al [33] offers a conceptual framework for identifying and addressing the challenges in technology-enabled solutions for family caregivers. While not yet a theoretical basis to study and analyze caregivers’ and care recipients’ use of technology, the conceptual framework by Lindeman et al [33] pointed to several factors that influence the caregivers’ technology adoption. The individual-level moderators involved user capacity and family, and socioeconomic moderators encompassed race and ethnicity, income, and geographic location [33]. Rather than directly assessing user capacity, we explored factors potentially linked to user capacity, such as age and education for caregivers, and cognitive dysfunction for care recipients.

## Methods

### Data Source and Study Population

This study collected cross-sectional data from a web-based survey about technology use among paid and unpaid caregivers of older adults who were recruited through a Qualtrics panel. To be eligible to participate in the web-based survey, the respondents must have provided 8 or more hours of weekly care for at least 1 adult care recipient aged 50 years or older (N=626). Recognizing that caregivers may be caring for multiple individuals, the caregiver was asked to respond to the survey questions in the context of the oldest person to whom they provided at least 8 hours of care. Quota sampling was used to ensure data were collected from a diverse sample reflecting the general characteristics of the US caregiving population.

Predetermined targets were set: 75% of the recruited sample were to be female, 50% at least 50 years old, and no more than 60% White. Geographic targets were also set to represent the regional population proportion (ie, 17.2% in the Northwest, 20.9% in the Midwest, 23.8% in the West, and 38.1% in the South [34]). This study focused on unpaid caregivers (n=486). The web-based survey commenced with a set of screening questions to identify eligible individuals. Those who were not screened out were provided with study information necessary for informed consent. Only those who agreed to participate were invited to complete the web-based survey.

This study differentiated between technology devices and technology functions. The respondents were asked whether they used each of the 7 devices (ie, cell phone, smartphone, tablet, computer, e-reader, voice-activated assistant, and wearable or smartwatch for activity tracking) and 8 functions (ie, communication, ride-sharing, online shopping, online banking, navigation, online entertainment, medication alerts or tracking, and physical activity tracking). The types of devices and functions were determined based on the 2020 AARP *Tech and the 50+ Survey* report [25]. We dropped some of the minimally used devices (eg, virtual reality device, 1%) and combined functions (eg, instead of individually assessing games, music, and video or movie streaming, they were consolidated into the “online entertainment” category) [25]. The respondents were also asked about their oldest care recipients’ use of the same devices and functions. The total numbers of devices (ranging from 0 to 7) and functions (ranging from 0 to 8) used were calculated separately for caregivers and care recipients.

Socioeconomic and demographic characteristics of the respondents were collected using the web-based survey: age (years), sex (male or female), race and ethnicity (Non-Hispanic White or others), household income (less than US $50,000 or US $50,000 or more), and education (high school graduate and lower educational attainment or higher). The web-based survey also asked about the respondents’ place of residence (zip code), and rural-urban commuting area codes were used to classify the place of residence into rural or urban areas. The respondents were also asked about their oldest care recipient’s age and place of residence (rural or urban areas classified based on the care recipient’s zip code). Respondents who reported being aware of their care recipient’s chronic conditions were also asked about their care recipients’ cognitive dysfunction (eg, dementia) and sensory impairment (eg, severe vision or hearing problems).

### Statistical Analysis

Frequencies and percentages or means and SDs were used to describe the caregivers’ and their care recipients’ background information and use of technology devices and functions. Cohen κ statistics were estimated to examine the concordance of technology use among caregivers and care recipients. The magnitude of matching was classified into poor (κ<0.20), fair (κ=0.21-0.40), moderate (κ=0.41-0.60), good (κ=0.61-0.80), and very good (κ=0.81-1.00) matching categories. Along with Cohen κ coefficient estimation, the McNemar test was performed to compare the marginal proportions of caregivers and care recipients using or not using each technology. Next, separate multivariable Poisson regression analyses were performed to predict the total number of devices and functions used among caregivers based on caregivers’ age, sex, race and ethnicity, household income, education, and place of residence. Separate multivariable Poisson regression analyses were performed to predict the total number of devices and functions used among care recipients based on the care recipients’ age, place of residence, cognitive dysfunction, and sensory impairment and the total number of devices and functions used among caregivers. Only 438 (90.1%) out of 486 respondents were aware of their care recipients’ chronic conditions; therefore, the regression models for predicting care recipients’ technology use included a smaller sample size than the regression models for predicting caregivers’ technology use. All statistical analyses were performed using SAS 9.4 (SAS Institute Inc), and a significance level of .05 was used.

### Ethical Considerations

The informed consent document was integrated at the outset of the web-based survey, and only those who agreed to participate proceeded to the subsequent sections of the survey. Given the web-based nature of the study, a waiver of documentation of informed consent was requested. Upon the completion of the study, any personally identifiable information (zip code) was deleted and age was truncated to 90 years old. Within Qualtrics, participant stipends were integrated into the survey, and each participant received a stipend upon the completion of the study. Based on the estimate provided Qualtrics, each participant was paid between US $7 and US $8. The study has been reviewed and approved by the Texas A&M University Institutional Review Board (IRB2019-1128M).

## Results

### Study Participants

The average age was 60.8 (SD 12.11) years for caregivers and 74.9 (SD 11.61) years for their oldest care recipient (Table 1). The majority of caregivers were female (363/485, 74.8%) and non-Hispanic White (331/483, 68.5%). Nearly 50% (241/486) had a household income less than US $50,000, and 20.6% (100/486) had high school or lower educational attainment. In all, 9.1% (44/483) of caregivers and 9.5% (46/482) of care recipients resided in rural areas. Of the 438 care recipients with available information, 43.2% (n=189) had cognitive dysfunction and 32.9% (n=144) had sensory impairment. On average, the caregivers used 3.4 devices and 4.2 functions, and their oldest care recipients used 1.8 devices and 1.6 functions.

**Table 1.** Characteristics of caregivers and care recipients and their technology use (n=486).

Characteristics	Caregivers	Care recipients
Age (y), mean (SD)	60.8 (12.11)	74.9 (11.61)
Female, n/N (%)	363/485 (74.8)	N/A^a^
Non-Hispanic White, n/N (%)	331/483 (68.5)	N/A
Household income less than US $50,000, n/N (%)	241/486 (49.6)	N/A
High school or lower educational attainment, n/N (%)	100/485 (20.6)	N/A
Rural residence, n/N (%)	44/483 (9.1)	46/482 (9.5)
Having cognitive dysfunction, n/N (%)^b^	N/A	189/438 (43.2)
Having sensory impairment, n/N (%)^b^	N/A	144/438 (32.9)
Number of devices used, mean (SD)^c^	3.4 (1.35)	1.8 (1.49)
Number of functions used, mean (SD)^d^	4.2 (1.73)	1.6 (1.92)

a N/A: not available.

b Cognitive function and sensory impairment information was only available for care recipients and reported by 556 (88.8%) out of 626 total eligible caregivers.

c Number of devices used ranged from 0 to 7.

d Number of functions used ranged from 0 to 8.

### Comparing Technology Use Among Caregivers and Care Recipients

McNemar tests showed that significantly greater proportions of caregivers used all examined technologies than their care recipients (all *P*<.05), with the exception of the medication alerts or tracking function (*P*=.45; Table 2). κ coefficients ranged from 0.09 to 0.42 (Table 3), indicating a poor to moderate degree of matching (ie, concurrent use or no use) of technology among caregivers and care recipients. For example, there were 84% (404/481) of dyads in which the caregiver used a computer, yet there were only 31.2% (150/481) of dyads in which both the caregiver and care recipient used a computer.

**Table 2.** Use of different devices and functions among caregivers and care recipients (n=486).

Variables		Caregivers, n/N (%)	Care recipients, n/N (%)	*P* value^a^
**Devices**				
	Cell phone	268/481 (55.7)	231/481 (48)	.002
	Smartphone	396/480 (82.5)	209/480 (43.5)	<.001
	Tablet	263/481 (54.7)	112/481 (23.3)	<.001
	Computer	404/481 (84)	160/481 (33.3)	<.001
	E-reader	98/480 (20.4)	44/480 (9.2)	<.001
	Voice-activated assistant	143/481 (29.7)	74/481 (15.4)	<.001
	Wearables for activity tracking	73/481 (15.2)	34/481 (7.1)	<.001
**Functions**				
	Communication	407/481 (84.6)	178/481 (37)	<.001
	Ride-sharing	95/480 (19.8)	29/480 (6)	<.001
	Online shopping	393/481 (81.7)	133/481 (27.7)	<.001
	Online banking	358/480 (74.6)	119/480 (24.8)	<.001
	Navigation	333/479 (69.5)	89/479 (18.6)	<.001
	Online entertainment	271/480 (56.5)	128/480 (26.7)	<.001
	Medication alerts or tracking	56/481 (11.6)	50/481 (10.4)	.45
	Physical activity tracking	92/481 (19.1)	39/481 (8.1)	<.001

a *P* value from the McNemar test examining whether there is a statistically significant difference in the proportions of caregivers and care recipients using or not using each technology.

**Table 3.** Use and nonuse of different devices and functions in both caregivers and care recipients (n=486).

Variables		Use by both caregivers and care recipients, n/N (%)	Nonuse by both caregivers and care recipients, n/N (%)	Cohen κ coefficient^a^
**Devices**				
	Cell phone	180/481 (37.4)	162/481 (33.7)	0.42
	Smartphone	194/480 (40.4)	69/480 (14.4)	0.17
	Tablet	87/481 (18.1)	193/481 (40.1)	0.20
	Computer	150/481 (31.2)	67/481 (13.9)	0.11
	E-reader	26/480 (5.4)	364/480 (75.8)	0.27
	Voice-activated assistant	58/481 (12.1)	322/481 (66.9)	0.42
	Wearables for activity tracking	16/481 (3.3)	390/481 (81.1)	0.22
**Functions**				
	Communication	168/481 (34.9)	64/481 (13.3)	0.12
	Ride-sharing	20/480 (4.2)	376/480 (78.3)	0.25
	Online shopping	127/481 (26.4)	82/481 (17)	0.12
	Online banking	105/480 (21.9)	108/480 (22.5)	0.11
	Navigation	75/479 (15.7)	132/479 (27.6)	0.09
	Online entertainment	122/480 (25.4)	203/480 (42.3)	0.39
	Medication alerts or tracking	22/481 (4.6)	397/481 (82.5)	0.34
	Physical activity tracking	20/481 (4.2)	370/481 (76.9)	0.22

a Poor (κ>0.20), fair (κ=0.21-0.40), moderate (κ=0.41-0.60), good (κ=0.61-0.80), and very good (κ=0.81-1.00) matching.

### Correlates of Technology Use Among Caregivers

The multivariable Poisson regression analyses suggested that the expected number of devices used among caregivers with household incomes less than US $50,000 was 0.88 times the number of devices used among those with household incomes of US $50,000 or more (*P*=.02; Table 4). Using a separate regression analysis, the results indicated that the adjusted mean number of functions used among caregivers decreased by 0.8% for every 1-year increase in the caregivers’ age (*P*<.001). Additionally, the adjusted mean number of functions used among caregivers was negatively associated with household income (b=−0.097; *P*=.04) and educational attainment (b=−0.188; *P*=.002). The estimated number of functions used among caregivers in the lower household income and lower educational attainment categories was significantly lower than that of caregivers with higher socioeconomic status.

**Table 4.** Multivariable Poisson regression analysis for predicting the total number of devices and functions used among caregivers (n=486).

Outcome and variables		b^a^ (SE)		IRR^b^ (95% CI)	*P* value
**Number of devices**					
	Age	0.001 (0.002)		1.001 (0.997-1.005)	.67
	Female	0.035 (0.059)		1.036 (0.923-1.162)	.55
	Non-Hispanic White	−0.026 (0.054)		0.975 (0.877-1.083)	.63
	Household income less than US $50,000	−0.130 (0.053)		0.878 (0.791-0.975)	.02
	High school or lower educational attainment	−0.086 (0.066)		0.918 (0.806-1.045)	.20
	Rural residence	−0.079 (0.094)		0.924 (0.769-1.110)	.40
**Number of functions**					
	Age	−0.008 (0.002)		0.992 (0.989-0.996)	<.001
	Female	−0.017 (0.053)		0.983 (0.886-1.090)	.74
	Non-Hispanic White	−0.046 (0.048)		0.955 (0.869-1.049)	.34
	Household income less than US $50,000	−0.097 (0.048)		0.908 (0.826-0.998)	.04
	High school or lower educational attainment	−0.188 (0.061)		0.829 (0.735-0.934)	.002
	Rural residence	−0.068 (0.085)		0.934 (0.792-1.103)	.42

a b: regression coefficient.

b IRR: incidence rate ratio.

### Correlates of Technology Use Among Care Recipients

For care recipients, age and cognitive dysfunction were negatively associated with number of devices (b=−0.024; *P*<.001 and b=−0.394; *P<*.001, respectively) and functions (b=−0.032; *P*<.001 and b=−0.370; *P*=.002, respectively; Table 5). In contrast, caregivers’ technology use was positively associated with care recipients’ technology use (b=0.184; *P*<.001 for devices and b=0.238; *P*<.001 for functions; Table 5).

**Table 5.** Multivariable Poisson regression analysis for predicting the total number of devices and functions used among care recipients (n=438).

Outcome and variables		b^a^ (SE)		IRR^b^ (95% CI)	*P* value
**Number of devices**					
	Age	−0.024 (0.003)		0.976 (0.970-0.983)	<.001
	Rural residence	−0.102 (0.125)		0.903 (0.707-1.154)	.41
	Cognitive dysfunction	−0.394 (0.080)		0.675 (0.577-0.789)	<.001
	Sensory impairment	0.002 (0.082)		1.002 (0.854-1.176)	.98
	Number of devices used by caregiver	0.184 (0.027)		1.202 (1.142-1.267)	<.001
**Number of functions**					
	Age	−0.032 (0.005)		0.969 (0.960-0.979)	<.001
	Rural residence	−0.069 (0.186)		0.934 (0.648-1.344)	.71
	Cognitive dysfunction	−0.370 (0.121)		0.691 (0.545-0.876)	.002
	Sensory impairment	−0.038 (0.123)		0.963 (0.756-1.225)	.76
	Number of devices used by caregiver	0.238 (0.033)		1.269 (1.189-1.354)	<.001

a b: regression coefficient.

b IRR: incidence rate ratio.

## Discussion

### Principal Findings

Based on the data analysis and results analyzed in the above section, some key findings provide a fuller and more specific understanding of older adults’ and their caregivers’ use of digital technologies and highlight the contextual factors that may either affect resistance or enhance accessibility and receptivity of technology-based interventions in a broader caregiver population.

Key findings and reflections on current and parallel research are presented below.

### Use of Technology and Comparison

This study examined unpaid caregivers’ use of various technology and their reports of their older adult care recipient’s use. It was observed that a greater proportion of caregivers reported using technologies themselves than being used by their care recipients. In our study, although 82.5% (396/480) and 84% (404/481) of caregivers reported using smartphones and computers, only 43.5% (209/480) and 33.3% (160/481) of older adults used smartphones and computers, respectively. This implies greater access to technology by caregivers than their older adult care recipients. Furthermore, although caregivers reported a higher number of functions used than the number of device types used (eg, 3.4 devices and 4.2 functions), their older care recipients reported a fewer number of functions than the number of device types used (eg, 1.8 devices and 1.6 functions). These findings imply that caregivers are more likely to maximize the potential of a technology than their care recipients, who are typically older and in poorer health [20,21]. Our findings support the 2020 AARP tech trend report [5], which pointed out that despite older adults’ high engagement with their devices, “many are not using the technology to its full potential.” For example, fewer than half of smart home assistant or smart speaker owners used the device daily according to the 2019 national survey [5]. Although the proportion increased to about 57% in 2023 [35], the rate has still remained relatively low. The value of technology in enabling aging in place and reducing caregiver burden will only become further enhanced when these devices can be used to their fullest potential by older adults.

The age-related discrepancy in technology adoption is likely to be associated with skills in using technology but also with attitudes related to technology. For example, “perceived needs” is an important attitudinal factor in behavior adoption [36]. In this study, there was the lack of statistically significant differences between caregivers and their care recipients in medication management technology. These findings are consistent with research by both Abrashkin et al [28] and Portz et al [37], who also found that older adults in an advanced illness management program had significantly less access to and confidence in using technology (eg, computer, internet, tablet, and cell phone) than the program enrollees’ caregivers, except for medical alert devices such as medication management systems [28,37]. While the reasons underlying these findings have not been fully examined yet, this could be related to a similar level of perceived needs by both parties.

### Correlates of Technology Use in Caregivers

Among caregivers, older age and lower socioeconomic factors (household income and education) were negatively associated with the number of technological devices or functions used. This finding is consistent with extant literature [38-41]. Our study further explores this relationship by revealing a difference between devices and functions. Although the number of technological devices used by caregivers was only significantly associated with household income, the number of technological functions in use was associated with multiple factors (ie, age, household income, and education).

Regarding ownership or access to technological devices, the age and socioeconomic aspects of the digital divide seem to be narrowing [5]. However, the age and socioeconomic aspects of the digital divide appear to remain in technology use [42]. Among the more critical issues facing many older adults in using digital devices such as smartphones and tablets, they lack the basic digital literacy required to use multimedia interactive devices with touchscreen technology [43,44], and their digital literacy level is likely to diminish with age [35]. Assumptions in the past were that if access to devices and basic training were provided, the “grey span” of the digital divide could be eliminated. For older adults, each new operating system revision or interface for existing devices can be a traumatic event, as what worked before no longer does. Cao et al [45] described the information overload and system feature overload of new digital applications that resulted in increased fatigue and technostress of the older adult users, further increasing their resistance to technology adoption. These findings align with the AARP national survey findings indicating a limited set of tasks performed by older adults on technology [5,35]. This continuing of the digital divide can disproportionately impact caregiving for older care recipients, especially those whose primary caregivers are often older adults.

### Correlates of Technology Use in Care Recipients

Our study found that, for care recipients, age and cognitive dysfunction were negatively associated with the number of devices and functions. In contrast, care recipients’ use of a device or function was positively associated with the use of the technology among their caregivers. Along with the previous findings about the correlates of technology use in caregivers, these findings align with Baishya and Samalia’s [46] assertion that technology adoption is contextual. This study’s findings emphasize the need for additional research to identify and understand the contextual factors to enhance the accessibility and receptivity of technology-based interventions in a broader aging community.

### Limitations

This study is not without limitations. Efforts were made to diversify the convenience Qualtrics panel sample by specifying the proportion of sample characteristics of respondents to ensure heterogeneity in key factors such as age, gender, race and ethnicity, and geographic region. However, this study did not use probability sampling and is subject to a potential nonprobability sampling bias. For example, given the nature of web-based recruitment, the study population already has access to the internet. Although the majority of caregivers have access to the internet (eg, 78% in 2018 [47]), the study findings may not be generalizable to a group of caregivers without access to the internet. Additionally, this study used proxy responses for care recipients’ technology use (ie, as reported by their caregivers). Further, some key sociodemographic characteristics of care recipients were not collected.

Our restriction to unpaid caregivers who provided at least 8 weekly hours of care was intended to ensure that caregivers were familiar with their care recipients. However, it is important to note that previous literature indicates that caregivers tend to underestimate care recipients’ physical and cognitive functions and certain activities [48,49] and may also be imprecise in reporting technology use. In this study, many care recipients had cognitive impairments or sensory impairments, which might have adversely affected caregivers’ perception of care recipients’ user capacity and actual use of technology. However, we also note that many older persons do experience these functional limitations and impairments and their inclusion is important for reflecting health conditions and technology use in this population, albeit recognizing reporting limitations. This study was not able to differentiate the cross-use or whether the technology was used explicitly for caregiving functions. Future studies can benefit from the specification of the purpose of different technologies related to caregiving tasks and more precise measurement of care recipients’ technology use, such as daily diary use of technology devices and functional use over a specified period of time or digital tracking of technology use.

This study was conducted prior to the onset of the COVID-19 pandemic; hence, it does not reflect what might have changed in attitude toward or use of technologies, as well as the evolution of technologies. However, it differentiates between technology use and function among both caregivers and care recipients and provides important insights related to disparities in access to technology, which was a critical factor in access to health care and other social services during the COVID-19 pandemic. Furthermore, despite the increase in technology use in older adults since the COVID-19 pandemic, this study’s findings align with the more recent report on older adults’ technology use pattern regarding the use of functions and socioeconomic correlates (eg, income) [35].

### Conclusions

With the increasing use of technology solutions for caregiving that are becoming available on the market, it is important to be aware of factors associated with the current digital divide in technology use—both in terms of the number and diversity of devices and their functional use. It is critical to look forward to what the future might hold regarding the technology being used to reduce caregiver burden and enhance care recipients’ health, independence, and quality of life. A digital divide among older adults can exacerbate greater health disparity since technology is a powerful source for obtaining information and communicating with health care and social service providers [50].

A major finding from this study was the existence of significant disparities in the use of technological devices and functions among caregivers and their older adult care recipients. Among caregivers, significant differences were observed in technology use based on age and socioeconomic factors. In addition, this study suggested that caregivers’ technology use is an enabling factor for older care recipients’ technology use, independent of advanced age and cognitive impairment, which depressed use, indicating pathways for clinical intervention.

This study was conducted before the onset of the COVID-19 pandemic, which demonstrated the growing importance of connecting on the web for basic health care. The extent to which observed relationships between individual and socioeconomic moderators and technology use have changed since COVID-19 is a question for further study. The importance of technology use has become more salient during the recommended “physically distant stay at home” orders for older adults to stay socially connected with loved ones or professional social connectors, whether living at home, in assisted living facilities, or even nursing homes [51]. Additionally, familiarity with or access to technology can facilitate or act as a barrier to obtaining COVID-19 vaccinations. For example, the reach of a digital platform to track vaccinations and make follow-up interactions among older adult populations, who would benefit greatly from such technology, will depend largely upon the extent telecommunication or telehealth is used or accepted by older adults or their caregivers.

In summary, this study adds to the rapidly expanding field of technology in the health and aging realm by describing potential contextual factors in technology use, which may contribute to the disparities in technology use among older adults and their care recipients [52,53]. Further efforts are needed to expand the understanding of how these contextual factors contribute to technology adoption among caregivers and their care recipients and the benefits and costs of such technological innovations [54]. Especially relevant is how social workers, health professionals, educators, and the community can facilitate and maintain appropriate use of new and emerging technology for critical interactions normally and enable access to the needed caregiver and social resources during the COVID-19 pandemic or after it subsides. Furthermore, future research could gain additional benefits by concentrating on broader categories of functions. This approach would enable a more targeted investigation into particular functions related to specific outcomes, such as economic functions and financial health.
